# Long-term Prognostic Value of Estimated Plasma Volume in Heart Failure with Preserved Ejection Fraction

**DOI:** 10.1038/s41598-019-50427-2

**Published:** 2019-10-07

**Authors:** Chen-Yu Huang, Ting-Tse Lin, Yi-Fan Wu, Fu-Tien Chiang, Cho-Kai Wu

**Affiliations:** 1 Division of Cardiology, Department of Internal Medicine, National Taiwan University College of Medicine and Hospital, Taipei, Taiwan; 2 Division of Cardiology, Department of Internal Medicine, Kinmen Hospital, Ministry of Health and Welfare, Kinmen, Taiwan; 3 Division of Cardiology, Department of Internal Medicine, National Taiwan University College of Medicine and Hospital Hsin-Chu Branch, Hsin-Chu City, Taiwan; 4 Department of Biological Science and Technology, National Chiao Tung University, Hsinchu, Taiwan; 5 Department of Family Medicine, Taipei City Hospital, Renai Branch, Taipei, Taiwan

**Keywords:** Cardiac hypertrophy, Heart failure

## Abstract

Plasma volume, estimated by several indirect methods, has been viewed as a biological surrogate for intravascular fluid status. The clinical implication of estimated plasma volume status (ePVS) for long term outcomes in heart failure with preserved ejection fraction (HFpEF) remains unclear. We investigate the prognostic value of ePVS calculated by Strauss formula and its association with cardiovascular events and mortality in a prospective HFpEF cohort. There were 449 individuals met the inclusion criteria of our cohort. Estimated plasma volume variation (ΔePVS) and its instantaneous derivatives were calculated by the Strauss formula. Our study endpoints were events of heart failure hospitalization and mortality. Kaplan–Meier estimates and Cox regression analysis were applied to determine the power of ΔePVS and baseline ePVS in predicting long term cardiovascular outcomes. Both baseline ePVS and ΔePVS were independent predictors of heart failure hospitalization and mortality. Kaplan-Meier estimates of these outcomes stratified by optimal cut-off value showed that HFpEF individuals with higher baseline ePVS and ΔePVS were associated with elevated risk of composite endpoint of heart failure hospitalization and mortality. This study demonstrated the prognostic value of a novel biological surrogate, instantaneous derivatives ePVS, in predicting long term cardiovascular outcomes in HFpEF population. Monitoring instantaneous plasma volume may assist in identifying patients at high risk for future cardiovascular events. Further prospective studies validating the role of ePVS in predicting long-term prognosis in patients with HFpEF are warranted.

## Introduction

The prevalence of heart failure hospitalization in heart failure with preserved ejection fraction (HFpEF) has increased over time^1^. However, the pathophysiology of HFpEF is heterogeneous and remains incompletely defined. Emerging models have suggested that proinflammatory cardiovascular and non-cardiovascular conditions lead to systemic microvascular endothelial inflammation, global cardiac remodeling, impaired coronary flow reserve, and subsequent myocardial fibrosis^2,3^. However, medical therapies that improve outcomes in patients with heart failure with reduced ejection fraction (HFrEF) have not shown convincing benefit in HFpEF, especially in regards to composite end points of all-cause or cardiovascular mortality and heart failure hospitalization^4–6^.

Congestion is a major reason for hospitalization in patients with HFpEF and is primarily managed with diuretics or ultrafiltration. Nevertheless, no standardized definition of adequate decongestion is currently available to guide clinical practice. In addition, traditional markers of decongestion such as dry body weight and fluid loss show poor correlation with symptom relief and/or long-term outcomes^7^.

Persistent congestion at discharge is associated with increased re-hospitalization and mortality. On the other hand, achieving a balanced fluid status can lead to a better quality of life in heart failure patients^8,9^. Given the difficulty in precisely evaluating congestion in HFpEF, biomarkers and novel approaches are needed to guide clinicians in the management of congestion in order to prevent hospital readmissions.

Plasma volume (PV), assessed indirectly by several published methods, has been considered a novel biological surrogate for congestion and has been shown to be associated with post-discharge outcomes^10^. Recently, congestion assessment by estimated plasma volume status (ePVS) and its instantaneous derivate has been shown to predict post-discharge cardiovascular events in HFrEF^11^. However, no study has shown an association between ePVS and outcomes in HFpEF population. Ventricular diastolic dysfunction, whether present at rest or induced by stress, is a central perturbation in HFpEF and can cause increased sensitivity to changes in volume load. The goal of this study was to investigate the prognostic value of ePVS and its association with heart failure hospitalization and mortality using a prospective cohort of Asians with HFpEF.

## Material and Methods

### Study subjects

This study complies with the Declaration of Helsinki and was approved by the institutional review board of the National Taiwan University Hospital. All individuals gave their written informed consent prior to participation in the study.

Patients fulfilling diagnostic criteria of HFpEF were enrolled between July 1995 to October 2016. The diagnosis of HFpEF was defined by: (1) symptoms of exertional dyspnea (i.e., New York Heart Association [NYHA] functional class II-III); (2) heart failure based on the Framingham criteria and preserved systolic function (ejection fraction ≥ 50%); and (3) echocardiographic evidence of LV diastolic dysfunction^12–15^. In this cohort, patients diagnosed with significant coronary artery disease (diameter stenosis ≥ 50%) by coronary angiography were excluded. Patients in end-stage renal disease or with significant hepatic disease, secondary hypertension, pericardial disease, severe valvular heart disease, cancer, chronic obstructive pulmonary disease, and/or chronic atrial fibrillation were excluded. Individuals who died or experienced cardiovascular events <60 days after enrollment were also excluded from this study.

**Figure 1:**
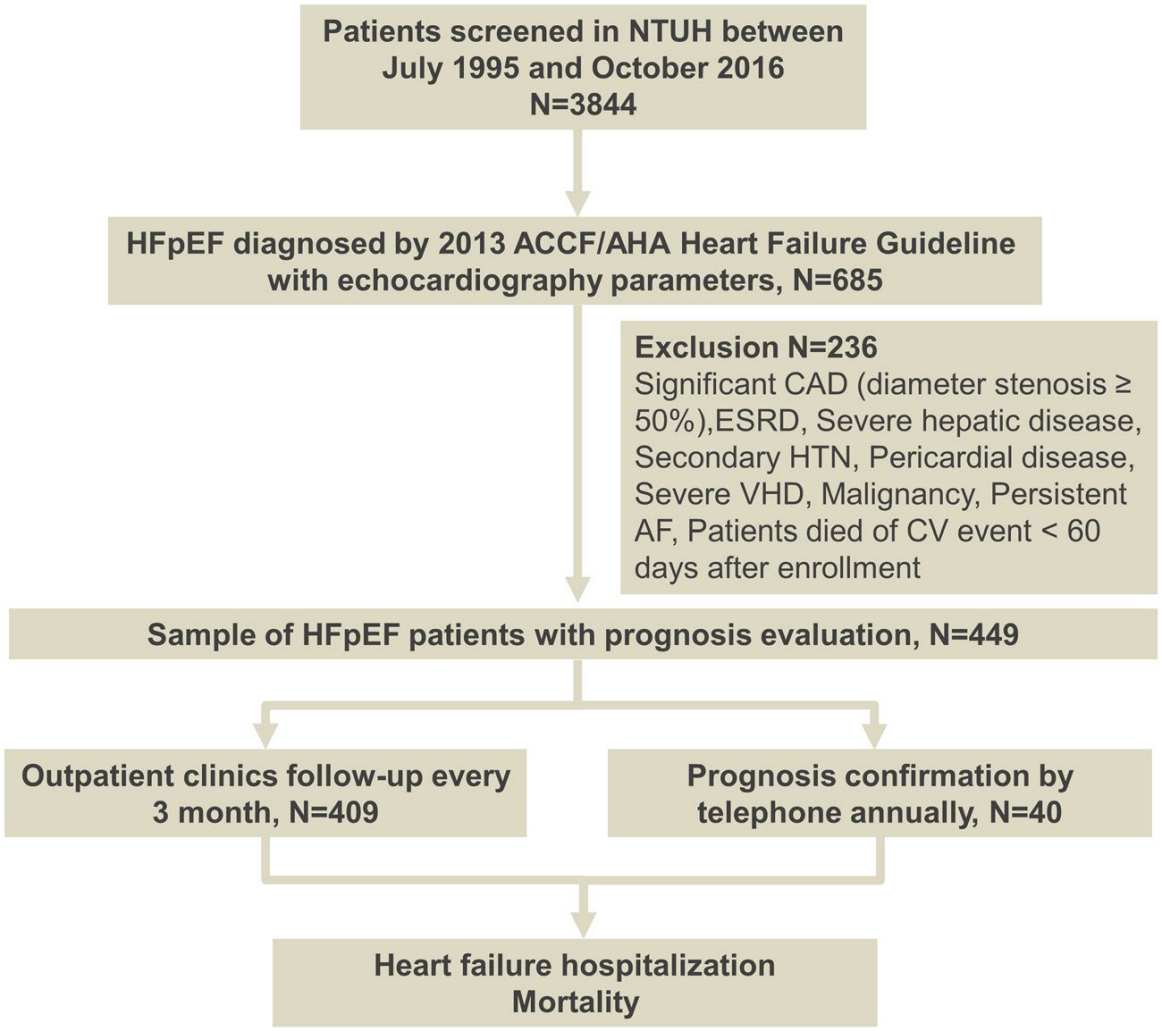
Flow chart of enrollment and follow-up of study cohort.

Of note, patients experiencing clinically detected bleeding events were excluded from the study since either blood loss or transfusion therapy would alter hemoglobin level, thereby influencing the calculation of plasma volume stated below. A flow chart for the inclusion and the follow-up algorithm is shown in Fig. 1.

Demographic data were collected from patient medical chart records. Hypertension was defined as a systolic blood pressure of ≥140 mmHg, a diastolic blood pressure of ≥90 mmHg, or use of at least one class of antihypertensive agents. Non-insulin-dependent diabetes mellitus was defined as a fasting blood glucose concentration >126 mg/dL and/or the use of at least one oral hypoglycemic agent. Information regarding medication, such as the use of angiotensin-converting-enzyme inhibitors (ACEI) and/or angiotensin II receptor blockers (ARB), calcium channel blockers (CCB), diuretics, nitrates and/or β-blockers was also recorded.

### Estimation of change in plasma volume

To estimate relative changes in plasma volume between enrollment and the occurrence of an event, the Strauss formula was employed using changes in hemoglobin and hematocrit concentrations between 2 time points^16^. The Strauss formula has been shown to be associated with cardiovascular events in previous study and is defined as follows^11^:

$$\Delta \mathrm{ePVS}=100\times \frac{hemoglobin(D0)}{hemoglobin\,(D1)}\times \frac{1-hematocrit(D1)}{1-hematocrit(D0)}-100$$

This formula can be interpreted as the relative change in estimated PV between 2 separate time points, D0 (enrollment) and D1 (date of event or at the end of follow-up). For this reason, instantaneous ePVS was defined as being proportional to this value. The instantaneous formula, at a given time for estimating PV as derived from ΔePVS, is calculated as:

$${\rm{I}}{\rm{n}}{\rm{s}}{\rm{t}}{\rm{a}}{\rm{n}}{\rm{t}}{\rm{a}}{\rm{n}}{\rm{e}}{\rm{o}}{\rm{u}}{\rm{s}}\,{\rm{e}}{\rm{P}}{\rm{V}}{\rm{S}}=\frac{1-hematocrit}{hemoglobin}\times 0.01$$

Therefore, by collecting the hemogram data for every individual at the time of enrollment. We acquired the baseline ePVS of every patient at the beginning of the follow up period.

### Study endpoints

The main endpoints of interest in our study were all-cause mortality and heart failure (HF) hospitalization.

### Follow-up

The follow-up period for the cohort ended on October 31, 2016. All patients visited our out-patient clinic at least every three months; otherwise, they were interviewed by telephone annually. All of the patients were carefully followed-up, and the longest follow-up period was 7,692 days.

Information regarding the study endpoint was documented in chart records and/or via telephone interviews. Hemoglobin and hematocrit data were obtained at the date of enrollment as D0. D1 represented the time point when the study endpoint (HF hospitalization or mortality) occurred or at the end of the follow up if an event did not occur. For each patient, the time to death or cardiovascular event(s) was calculated from the date of enrollment to the date on which the study endpoints occurred. If an event did not occur, the patient was censored at the end of the study.

### Statistical analysis

Continuous variables were expressed as mean values ± standard deviation (SD), whereas categorical variables were expressed as frequencies. Associations between categorical variables were tested by Pearson’s chi squared test. To test whether the data were normally distributed, the Kolmogorov-Smirnov test was applied. Comparisons between the data showing normal distribution were performed using the Student’s t-test or otherwise by the Mann-Whitney U test. Univariate Cox proportional hazards regression analysis was used to examine factors associated with HF hospitalization and all-cause mortality. Predictors in the multiple Cox model were selected from the set of variables that reached statistical significance on univariate analysis, via a forward selection procedure with a significance limit to enter the model set to 0.05.

The survival time was defined as the duration between enrollment and the occurrence of an event. Receiver operating characteristic (ROC) curves were applied to assess the discriminative power of baseline ePVS and the interval change of ePVS (ΔePVS) derived from the Strauss formula, thereby differentiating the composite endpoint of HF hospitalization and all-cause mortality. The optimal cutoff point, defined as that with the minimum value of (1-sensitivity)^2^ + (1-specificity)^2^, or the shortest distance from the left upper corner to the ROC curve, was reported. Afterwards, we divided our cohort in two groups by the reported optimal cutoff point of ΔePVS and baseline ePVS respectively. Kaplan-Meier method and log-rank test were adopted to compare the survival free from composite endpoint. Statistical analysis was performed using IBM SPSS Statistics version 21.0 (IBM, Armonk, New York, USA). Two-sided p values < 0.05 were considered to indicate statistical significance.

### Validation of ePVS derived from Strauss formula

Since ePVS calculated from Strauss formula was not validated in the HFpEF population, we retrospectively identified subjects with HFpEF who were hospitalized due to acute decompensated heart failure in our previous study^17^. Among 63 subjects with HFpEF, there were 40 patients hospitalized due to ADHF in recent two years. The baseline characteristics were listed in Supplemental Table 1. The baseline ePVS and ΔePVS were calculated and serum level of pro-BNP level and diuretics use were also recorded.

## Results

### Baseline characteristics

Overall, a total of 449 individuals with HFpEF were enrolled in the study. All patients were followed-up with until the end of the study. The median follow-up period was 3,896 days, and the longest follow-up period was 7,692 days. Characteristics of the Patients stratified by HF hospitalization or baseline ePVS level are shown in Tables 1 and 2.

**Table 1:** Characteristics of the Patients with and without Heart failure (HF) hospitalization.

	All Cohort N = 449	HF hospitalization N = 111 (24.7%)	No HF hospitalization N = 338	*p*-value
***Clinical Parameters***				
Age	66 (58–73)	71 (65–76)*	65 (56–70)	<0.001
Male sex, N (%)	249 (55.5)	63 (56.8)	186 (55)	0.826
Body mass index (kg/m^2^)	24.07 (21.31–27.02)	23.73 (21.16–26.04)	24.17 (21.32–27.32)	0.151
Hypertension	292 (65)	76 (68.5)	216 (63.9)	0.423
Hyperlipidemia	182 (40.5)	53 (47.7)	129 (38.2)	0.076
Diabetes mellitus	105 (23.4)	35 (31.5)*	70 (20.7)	0.027
NYHA Class II	261 (58)	68 (61.3)	193 (57.1)	0.506
NYHA Class III	188 (42)	43 (38.7)	145 (42.9)	
Hemoglobin (g/dL)	13.5 (12.4–14.3)	13.4 (12.2–14.3)	13.5 (12.5–14.3)	0.293
Hematocrit (%)	39.6 (37.0–42.3)	39.2 (35.5–42.2)	39.7 (37.5–42.3)	0.198
Creatinine clearance (ml/min)	50 (43–60)	46 (39–55)*	51 (44–63)	<0.001
ΔePVS	4.09 (−7.29–22.7)	7.74 (−5.41–29.16)*	2.68 (−8.02–18.95)	0.004
Baseline ePVS	4.5 (4.05–5.06)	4.56 (4.03–5.26)	4.47 (4.05–5.00)	0.233
***Medication***				
Antiplatelet	319 (70.9)	68 (61.3)	188 (55.6)	0.321
ACEI/ARB	155 (34.4)	25 (22.5)*	129 (38.2)	0.003
Beta-blockers	210 (46.6)	47 (42.3)	163 (48.2)	0.324
CCB	220 (48.8)	46 (41.4)	174 (51.5)	0.080
Diuretics	238 (52.8)	54 (48.6)	160 (47.3)	0.827
***Echocardiographic parameters***				
LVEF (%)	69 (63.5–75)	69 (62–74)	70 (64–75)	0.225
LVEDD (mm)	45.8 (42–49)	45 (41.4–50)	46 (42–48.8)	0.982
LVESD (mm)	27 (24.1–31)	27 (24–32)	27 (24.15–30.5)	0.559
IVS (mm)	11 (10–13)	12 (10–13)	11 (10–12.9)	0.077
LVPW (mm)	11 (10–12)	11 (10–13)	11 (10–12)	0.153
LAVI (ml/m^2^)	36 (32–39)	37 (33–41)*	35 (32–39)	0.010
LVMI (gm/m^2^)	207 (167–250)	210 (164–261)	205 (167–247)	0.297
E (cm/s)	67 (56–81)	71 (57–83)	66 (55.75–79.25)	0.083
A (cm/s)	84 (69–97)	84 (65–98)	84 (70–97)	0.300
E/A ratio	0.79 (0.67–0.92)	0.79 (0.67–1.13)	0.79 (0.68–0.92)	0.327

Values were n, median (interquartile range), or proportion (%).

*p < 0.05, p-values were derived from the chi-square test for categorical variables and nonparametric Mann-Whitney U test for continuous variables.

Abbreviations: NYHA = New York Heart Association; A = late mitral inflow velocity; ACEI = angiotensin-converting-enzyme inhibitors; ARB = angiotensin II receptor blockers; CCB = calcium channel blocker; E = early mitral inflow velocity; ePVS = estimated plasma volume (Strauss formula); LAVI = left atrium volume index; LVEDD = left ventricle end diastolic diameter; LVEF = left ventricle ejection fraction; LVESD = left ventricle end systolic diameter; IVS = interventricular septum; LAVI = left atrium volume index; LVMI = left ventricle mass index; LVPW = left ventricle posterior wall.

**Table 2:** Characteristics of the Patients according to baseline ePVS level.

	Low baseline ePVS (<4.5) N = 224	High baseline ePVS (>=4.5) N = 225	*p* value
***Clinical Parameters***			
Age	63 (55–68)	67 (60–76)	0.001
Male, N (%)	159 (71)	90 (40)	<0.001
Body mass index (kg/m^2^)	24.5 (21.39–26.86)	23.54 (21.21–27.21)	0.356
Hypertension	143 (63.8)	149 (66.2)	0.597
Hyperlipidemia	82 (36.6)	100 (44.4)	0.091
Diabetes mellitus	38 (17.0)*	67 (29.8)	0.001
NYHA Class II	181 (80.8)	80 (35.6)	<0.001
NYHA Class III	43 (19.2)	145 (64.4)	<0.001
Hemoglobin (g/dL)	14.3 (13.8–15)	12.4 (11.7–12.9)	<0.001
Hematocrit (%)	42.3 (41.2–43.9)	37 (34.2–38.7)	<0.001
Creatinine clearance (ml/min)	48 (43–58)	53 (44–64)	<0.001
ΔePVS	10.5 (−1.23–28.86)	−2.85 (−12.53–15.88)	<0.001
***Medication***			
Antiplatelet	122 (54.5)	134 (59.6)	0.276
ACEI/ARB	77 (34.4)	77 (34.2)	0.973
Beta-blockers	104 (46.4)	106 (47.1)	0.885
CCB	112 (50)	108 (48)	0.672
Diuretics	110 (49.1)	104 (46.2)	0.541
***Echocardiographic parameters***			
LVEF (%)	70 (64–75)	69 (63–75)	0.507
LVEDD (mm)	46 (42–48)	45 (42–49)	0.516
LVESD (mm)	27 (24.4–30.9)	27 (24–31)	0.723
IVS (mm)	12 (10–13)	11 (10–12)	0.043
LVPW (mm)	11 (10–12)	11 (10–12)	0.025
LAVI (ml/m^2^)	36 (32–40)	36 (32–39)	0.656
LVMI (gm/m^2^)	214 (171.3–256)	199 (162–250)	0.189
E (cm/s)	65 (54–79)	70 (56–82)	0.047
A (cm/s)	83 (67–96)	85 (71–98)	0.411
E/A ratio	0.79 (0.67–0.91)	0.80 (0.67–0.92)	0.484

Abbreviations: NYHA = New York Heart Association; A = late mitral inflow velocity; ACEI = angiotensin-converting-enzyme inhibitors; ARB = angiotensin II receptor blockers; CCB = calcium channel blocker; E = early mitral inflow velocity; ePVS = estimated plasma volume (Strauss formula); LAVI = left atrium volume index; LVEDD = left ventricle end diastolic diameter; LVEF = left ventricle ejection fraction; LVESD = left ventricle end systolic diameter; IVS = interventricular septum; LAVI = left atrium volume index; LVMI = left ventricle mass index; LVPW = left ventricle posterior wall.

The most common comorbidity was hypertension (65%), followed by hyperlipidemia (40.5%) and diabetes mellitus (23.4). The patients enrolled were predominantly in NYHA function class II (58%), followed by class III (42%). The medication prescribed the most was antiplatelet agents (70.9%) followed by diuretics (52.8%). Over one third of patients were taking ACEI or ARB, β-blockers or calcium channel blockers.

Individuals having HF hospitalization were found to be more elderly and diabetic while other comorbidities and NYHA class were comparable. Though baseline hemoglobin and hematocrit were similar to the event-free group, they had worse renal function indicated by lower creatinine clearance. The prescription of medication did not differ much except that patient with HF hospitalization had less ACEI or ARB use. Echocardiographic findings were comparable except that patients with HF hospitalization had greater LA volume index (Table 1).

Stratifying patients into high and low baseline ePVS groups, we observed that patients with high baseline ePVS were more elderly, female predominant, diabetic and mostly in NYHA class III. They had lower hemoglobin and hematocrit levels but higher creatinine clearance level. The medications and most echocardiographic features were comparable between two groups (Table 2).

### Estimated Plasma volume status and outcomes

Baseline ePVS and Δ ePVS are both independent predictors of HF hospitalization (baseline ePVS, HR: 1.305, 95% CI: 1.084–1.571; Δ ePVS, HR: 1.005, 95% CI:1.001–1.01). Other additional predictors associated HF hospitalization includes age, hyperlipidemia and lower creatinine clearance (Table 3). Overall mortaliy was significantly associated with higher baseline ePVS and Δ ePVS (baseline ePVS, HR: 1.861, 95% CI: 1.57–2.207; Δ ePVS, HR: 1.011, 95% CI:1.007–1.015). Clinical covariates including male sex, age and lower creatinine clearance were strongly correlated to mortality while hyperlipidemia and diabetes mellitus were not (Table 4). It is noteworthy that both BMI and NYHA class are not potent predictors for HF hospitalization and mortality in our HFpEF cohort.

**Table 3:** Predictors of Heart failure hospitalization as determined via univariate and multivariate Cox proportional hazards regression analyses.

	Univariate		Multivariate	
	Hazard ratios (95% CI)	*p* value	Hazard ratios (95% CI)	*p* value
Male sex	1.276 (0.875–1.859)	0.205		
Age	1.068 (1.044–1.091)	<0.001	1.052(1.027–1.077)	<0.001
Hypertension	1.124 (0.752–1.679)	0.568		
Diabetes	1.729 (1.158–2.581)	0.007	1.144 (0.743–1.760)	0.542
Hyperlipidemia	1.622 (1.115–2.360)	0.011	1.524 (1.040–2.234)	0.031
NYHA classification	0.896 (0.612–1.314)	0.575		
Body mass index (kg/m^2^)	0.958 (0.907–1.012)	0.128		
Creatinine clearance (ml/min)	0.941 (0.922–0.960)	<0.001	0.949 (0.930–0.969)	<0.001
Δ ePVS	1.007 (1.003–1.011)	<0.001	1.005 (1.001–1.010)	0.024
Baseline ePVS	1.292 (1.092–1.528)	0.003	1.305 (1.084–1.571)	0.005
LVEF	0.981 (0.973–1.024)	0.387		

*p*-values were derived from Cox regression analysis.

Abbreviations: CI = confidence interval; ePVS = estimated plasma volume (Strauss formula); LVEF = left ventricle ejection fraction.

**Table 4:** Predictors of Mortality as determined via univariate and multivariate Cox proportional hazards regression analyses.

	Univariate		Multivariate	
	Hazard ratios (95% CI)	*p* value	Hazard ratios (95% CI)	*p* value
Male sex	1.954 (1.248–3.061)	0.003	1.837 (1.203–2.806)	0.005
Age	1.055 (1.029–1.081)	<0.001	1.058 (1.032–1.085)	<0.001
Hypertension	0.707 (0.459–1.089)	0.115		
Diabetes	1.367 (0.874–2.138)	0.171		
Hyperlipidemia	1.442 (0.938–2.218)	0.096		
NYHA classification	1.446 (0.971–2.154)	0.070		
Body mass index (kg/m^2^)	1.015 (0.957–1.076)	0.615		
Creatinine clearance (ml/min)	0.924 (0.903–0.945)	<0.001	0.926 (0.904–0.948)	<0.001
Δ ePVS	1.014 (1.010–1.018)	<0.001	1.011 (1.007–1.015)	<0.001
Baseline ePVS	1.763 (1.462–2.126)	<0.001	1.861 (1.570–2.207)	<0.001
LVEF	1.001 (0.976–1.026)	0.952		

P-values were derived from Cox regression analysis.

Abbreviations: CI = confidence interval; ePVS = estimated plasma volume (Strauss formula); LVEF = left ventricle ejection fraction.

**Figure 2:**
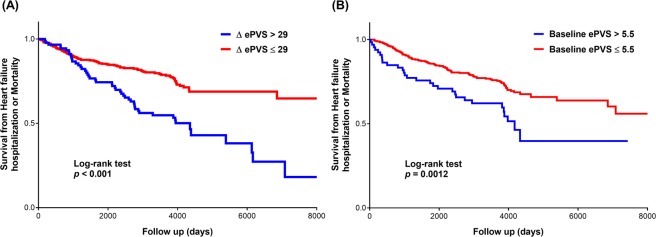
Kaplan–Meier estimates of HF hospitalization or Mortality outcomes stratified by plasma volume status represented by (**A**) ΔePVS or (**B**) baseline ePVS.

After a median follow-up period of 3,896 days, 97 patients (21.6%) died and 111 patients (24.7%) had HF hospitalization. Incidence of mortality and HF hospitalization were 23.5 and 45.6 per 1,000 patient-years respectively. Kaplan-Meier estimates of the composite endpoint of HF hospitalization and mortality stratified by optimal cut-off value showed that HFpEF individuals with higher baseline ePVS were predisposed to greater risk of HF hospitalization or mortality (log-rank test *p* = 0.0012). Likewise, patients with greater ΔePVS during follow-up period were associated with lower event-free survival from HF hospitalization or mortality (log-rank test *p* < 0.001) (Fig. 2A,B).

### Validation of ePVS derived from Strauss formula

We found delta ePVS was significantly correlated with NT-pro-BNP and diuretics dosage during hospitalization (Supplemental Table 2). These findings may suggest delta ePVS reflects the congestion status during acute decompensated stage of HFpEF.

## Discussion

Using a prospective HFpEF cohort over a long follow-up period, our results showed that higher ΔePVS was associated with both HF hospitalization and mortality. To the best of our knowledge, this is the first study to demonstrate the prognostic value of this new biological surrogate in predicting cardiovascular outcomes in HFpEF among Asian population. In addition, the instantaneous estimation of baseline ePVS was found to prognosticate future events of HF hospitalization and mortality based on multivariate Cox regression analysis. This baseline ePVS approach allows physicians to evaluate a patient’s congestive status and presage future cardiovascular events in addition to traditional routine clinical assessments and natriuretic peptide measurements.

### Major findings

The main reason for heart failure hospitalization is congestion, as manifested by dyspnea, orthopnea, and edema due to elevated LV filling pressure. Moreover, congestion can cause heart failure progression through further renin–angiotensin–aldosterone system (RAAS) and sympathetic activation, ventricular geometric changes, pulmonary hypertension, and other end-organ hypoperfusion.

In light of the difficulties in accurately evaluating volume status by clinical examination, various biomarkers and other novel approaches have been developed to help clinicians to quantify congestion. Natriuretic peptides have long been the most commonly used biomarkers of volume status. Some studies have also highlighted its combination with bioimpedance techniques to guide treatment in patients with acute decompensated heart failure in the emergency department^18^. In some studies, hemoconcentration (that occurs later during hospitalization despite worsening renal function) is associated with improved prognosis in acute heart failure.

Plasma volume (PV), the intravascular portion of the extracellular fluid volume, can be measured using standard dilutional analysis employed after the administration of tracer molecules. Nevertheless, it is feasible to indirectly estimate percentage shifts in PV (without measuring the absolute values from serial concomitant hemoglobin and hematocrit concentrations) using the Strauss formula^16^. Available data have demonstrated that PV changes have important implications in the clinical setting of heart failure management. Whereas PV appears to be expanded in edematous subjects before therapeutic intervention, it is contracted in patients with stable HF under conventional therapy^19^.

In our study, we observed that decongestion, as represented by a decrease in ΔePVS or baseline ePVS, was associated with better long-term prognosis. This finding corroborates data derived from four randomized trials in acute decompensated HF that reported a positive relationship between decongestion during hospitalization and post-discharge prognosis. In the post-hoc analysis of the PROTECT (Placebo-Controlled Randomized Study of the Selective Adenosine A1 Receptor Antagonist Rolofylline for Patients Hospitalized with Acute Decompensated Heart Failure and Volume Overload to Assess Treatment Effect on Congestion and Renal Function) trial, the EVEREST (Efficacy of Vasopressin Antagonism in Heart Failure Outcome Study With Tolvaptan) trial, and the ESCAPE (Evaluation Study of Congestive Heart Failure and Pulmonary Artery Catheterization Effectiveness) trial, hemoconcentration was viewed as a marker of decongestion and was associated with greater decongestion and decreased mortality and HF re-hospitalization rates^9,20,21^. Recently, post-hoc analysis of EPHESUS (Eplerenone Post-Acute Myocardial Infarction Heart Failure Efficacy and Survival Study) trial showed that plasma volume (as assessed by the Strauss formula and its instantaneous derived measurement) provided a good predictive value of early cardiovascular events in an HFrEF population^11^. Nevertheless, these studies were all conducted in patients with HFrEF and only short term outcomes were evaluated.

Diastolic dysfunction in HFpEF patients results in ineffective LA emptying and LV filling, reduced ability to enhance cardiac output with exercise, increases in pulmonary pressure, and contributes to the symptoms of congestion and fluid retention. Until now, no large randomized trial was available which proved the benefit of medication therapy on outcomes in HFpEF patients. Nevertheless, a previous published Treatment of Preserved Cardiac Function Heart Failure With an Aldosterone Antagonist (TOPCAT) trial which compared 886 patients randomized to spironolactone with 881 patients assigned to placebo showed that a composite primary outcome (HR: 0.82; 95% CI: 0.69–0.98), cardiovascular death (HR: 0.74; 95% CI: 0.57–0.97), and hospitalizations for HF (HR: 0.82; 95% CI: 0.67–0.99) were significantly reduced^22,23^. These results suggested that decongestion and maintaining fluid balance constitute the most important therapy in HFpEF patients and lead to a better prognosis.

Diuretics have been the mainstay of decongestion therapy but approaches to decongestion must be individualized based on a patient’s initial diuretic response, co-morbidity burden, and hemodynamic status. Given differences in evidence-based therapy guidelines, treatment for HFpEF differs from HFrEF, and such guidelines only recommend control of hypertension and diuretics directed toward relief of symptoms due to fluid overload^24,25^. Of note, instantaneous ePVS after treatment has been shown to be a better predictor of outcomes in patients with HFrEF and prior myocardial infarction^26^. Markers of decongestion are of paramount importance because residual or incomplete decongestion is associated with re-hospitalization and mortality. From the perspective of pathophysiology, patients with HFpEF are particularly sensitive to intravascular volume alteration. Our study provided a new approach for identifying patients at high risk for future cardiovascular events in an HFpEF population by using readily available biological variables, i.e., ΔePVS and instantaneous estimation of baseline ePVS.

The present study applied the Strauss formula to assess the severity of congestion in symptomatic patients with HFpEF and demonstrated that lower baseline ePVS was associated with better outcomes. Derived from our cohort analysis, a cut-off value of baseline ePVS above 5.5% represented fluid overload status and increased HF hospitalization and mortality. Hence, the measurement of baseline ePVS may help to tailor the diuretic dosage during hospitalization and also provide a useful prognostic indicator in the HFpEF population. The aim of our study is to utilize baseline ePVS and Δ ePVS to predict long-term rather than short-term cardiovascular events. Previous HF studies evaluating adverse event within the follow-up period varied from 12 weeks to 3 months. For example, the EuroHeart Failure survey program investigated the CV outcome among inpatients within 12 weeks follow-up. Organized Program to Initiate Lifesaving Treatment in Hospitalized Patients with Heart Failure (OPTIMIZE-HF) trials used endpoints of 60-day or 90-day mortality and HF re-admission^27,28^. Therefore, we intended to exclude individuals having cardiovascular events <60 days after enrollment to examine the long term effect of ePVS on mortality and HF hospitalization. In fact, in our cohort, there was no events of mortality or readmission for HF within 60 days after study enrollment.

Conceivably, the release of brain natriuretic peptide (BNP) is increased in heart failure, as ventricular cells are recruited to secrete this hormone in response to the high ventricular filling pressure^29^. Its inert metabolite, N-terminal pro-BNP (NT-proBNP), has been shown to be a good predictor of mortality and heart failure hospitalization in patients with HFpEF^30^. Nevertheless, variability in peptide measurements must be considered when interpreting serial BNP or NT-proBNP results^31^. Renal dysfunction, obesity, and pulmonary hypertension also have impacts on the change in measurement of natriuretic peptides^32,33^. Heart failure patients do not always receive medication, out-patient clinic treatment, or follow-up in the medical center where various biomarkers laboratory tests are available. In this situation, utilizing hematocrit to estimate PVS is a low-cost, easily-measurable alternative that is available in clinical practice. Repeat measurements and calculations of estimated PVS by checking the hematocrit could help physicians to adjust guideline-based medical therapy and predict outcomes in high risk patients.

### Limitations

Our study had several limitations. First, though indirect estimations of changes in plasma volume by the Strauss formula were previously validated in patients with scheduled plasma exchanges or ultrafiltration in acute decompensated heart failure^34,35^; no solid validation for its use has been reported in the HFpEF setting. Second, some other factors might have affected the estimation of plasma volume using the Strauss formula, including acute blood loss and conditions requiring frequent red blood cell transfusions. Although we excluded patients with anemia, gastrointestinal bleeding, and blood loss from the current study, other possible factors that could influence blood volume may have affected the estimation of plasma volume. In addition, the sample size in the current study was moderate, however, we followed patients with HFpEF in this cohort for more than 10 years. All patients were carefully followed for outcomes and this might have compensated for the limitation of small sample size. Lastly, our HFpEF cohort is comprised of Asians. The prognostic value of ePVS has been addressed in two other heart failure studies mainly from Caucasian. The first one evaluating heart failure with reduced ejection fraction complicating myocardial infarction showed that congestion assessed by the Strauss formula and an instantaneous derived measurement of plasma volume provided a predictive value of early cardiovascular events^11^. A recent study of plasma volume status published in 2019 also demonstrated that higher calculated estimates of PVS were independently associated with a higher risk of long-term clinical outcomes in HFpEF, and particularly, heart failure hospitalization^36^. Our findings in our HFpEF cohort are in consistence with the latter one with additional advantages that our study patients had longer follow-up period than the others. Therefore, our study is by far the only Asian cohort that investigate the value of ePVS in long term prognostic prediction among HFpEF population.

## Conclusions

In conclusion, this study demonstrated the prognostic value of a new biological surrogate, instantaneous estimation of baseline ePVS, in predicting HF hospitalization and mortaliy in HFpEF. Changes in plasma volume using the Strauss formula provide an easy method to monitor a patient’s plasma volume using only hemoglobin and hematocrit levels and may assist in identifying patients at high risk for future cardiovascular events. This method may also allow tailoring of diuretic doses and diet adjustments in the HFpEF population. Hence, further prospective studies investigating the role of plasma volume evaluation in long-term cardiovascular outcomes in HFpEF population are warranted.

## Supplementary information

Supplemental Tables

## References

[1] Steinberg BA (2012). Trends in patients hospitalized with heart failure and preserved left ventricular ejection fraction: prevalence, therapies, and outcomes. Circulation.

[2] Paulus WJ, Tschope C (2013). A novel paradigm for heart failure with preserved ejection fraction: comorbidities drive myocardial dysfunction and remodeling through coronary microvascular endothelial inflammation. J Am Coll Cardiol.

[3] Mohammed SF (2015). Coronary microvascular rarefaction and myocardial fibrosis in heart failure with preserved ejection fraction. Circulation.

[4] Wu, C. K. *et al*. *J Am Coll Cardiol*. **56**(23), 1930–6 (2010). 10.1016/j.jacc.2010.04.069 21109116

[5] Voors AA (2014). Spironolactone not effective in diastolic heart failure. Ned Tijdschr Geneeskd.

[6] Liu F (2014). Effects of beta-blockers on heart failure with preserved ejection fraction: a meta-analysis. PLoS One.

[7] Kociol RD (2013). Markers of decongestion, dyspnea relief, and clinical outcomes among patients hospitalized with acute heart failure. Circ Heart Fail.

[8] Mentz RJ (2014). Decongestion in acute heart failure. Eur J Heart Fail.

[9] Testani JM, Chen J, McCauley BD, Kimmel SE, Shannon RP (2010). Potential effects of aggressive decongestion during the treatment of decompensated heart failure on renal function and survival. Circulation.

[10] Testani JM (2013). Timing of hemoconcentration during treatment of acute decompensated heart failure and subsequent survival: importance of sustained decongestion. J Am Coll Cardiol.

[11] Duarte K (2015). Prognostic Value of Estimated Plasma Volume in Heart Failure. JACC Heart Fail.

[12] Wu CK (2011). Plasma levels of tumor necrosis factor-alpha and interleukin-6 are associated with diastolic heart failure through downregulation of sarcoplasmic reticulum Ca2+ ATPase. Crit Care Med.

[13] Wu CK (2014). Connective tissue growth factor and cardiac diastolic dysfunction: human data from the Taiwan diastolic heart failure registry and molecular basis by cellular and animal models. Eur J Heart Fail.

[14] Wu CK (2015). Galectin-3 level and the severity of cardiac diastolic dysfunction using cellular and animal models and clinical indices. Sci Rep.

[15] Paulus WJ (2007). How to diagnose diastolic heart failure: a consensus statement on the diagnosis of heart failure with normal left ventricular ejection fraction by the Heart Failure and Echocardiography Associations of the European Society of Cardiology. Eur Heart J.

[16] Strauss MB, Davis RK, Rosenbaum JD, Rossmeisl EC (1951). Water diuresis produced during recumbency by the intravenous infusion of isotonic saline solution. J Clin Invest.

[17] Wu CK (2017). Evolutional change in epicardial fat and its correlation with myocardial diffuse fibrosis in heart failure patients. J Clin Lipidol.

[18] Di Somma S (2010). Use of BNP and bioimpedance to drive therapy in heart failure patients. Congest Heart Fail.

[19] Kalra PR, Anagnostopoulos C, Bolger AP, Coats AJ, Anker SD (2002). The regulation and measurement of plasma volume in heart failure. J Am Coll Cardiol.

[20] van der Meer P (2013). The predictive value of short-term changes in hemoglobin concentration in patients presenting with acute decompensated heart failure. J Am Coll Cardiol.

[21] Greene SJ (2013). Haemoconcentration, renal function, and post-discharge outcomes among patients hospitalized for heart failure with reduced ejection fraction: insights from the EVEREST trial. Eur J Heart Fail.

[22] Pitt B (2014). Spironolactone for heart failure with preserved ejection fraction. N Engl J Med.

[23] Pfeffer MA, Braunwald E (2016). Treatment of Heart Failure With Preserved Ejection Fraction: Reflections on Its Treatment With an Aldosterone Antagonist. JAMA Cardiol.

[24] Yancy CW (2013). 2013 ACCF/AHA guideline for the management of heart failure: a report of the American College of Cardiology Foundation/American Heart Association Task Force on Practice Guidelines. J Am Coll Cardiol..

[25] Ponikowski P (2016). 2016 ESC Guidelines for the diagnosis and treatment of acute and chronic heart failure: The Task Force for the diagnosis and treatment of acute and chronic heart failure of the European Society of Cardiology (ESC)Developed with the special contribution of the Heart Failure Association (HFA) of the ESC. Eur Heart J..

[26] Kinnunen P, Vuolteenaho O, Ruskoaho H (1993). Mechanisms of atrial and brain natriuretic peptide release from rat ventricular myocardium: effect of stretching. Endocrinology..

[27] Cleland JG (2000). The Euro Heart Failure Survey of the EUROHEART survey programme. A survey on the quality of care among patients with heart failure in Europe. The Study Group on Diagnosis of the Working Group on Heart Failure of the European Society of Cardiology. The Medicines Evaluation Group Centre for Health Economics University of York. Eur J Heart Fail.

[28] Fonarow GC (2004). Organized Program to Initiate Lifesaving Treatment in Hospitalized Patients with Heart Failure (OPTIMIZE-HF): rationale and design. Am Heart J.

[29] Anand IS (2011). Prognostic value of baseline plasma amino-terminal pro-brain natriuretic peptide and its interactions with irbesartan treatment effects in patients with heart failure and preserved ejection fraction: findings from the I-PRESERVE trial. Circ Heart Fail..

[30] Jhund PS (2015). Changes in N-terminal pro-B-type natriuretic peptide levels and outcomes in heart failure with preserved ejection fraction: an analysis of the I-Preserve study. Eur J Heart Fail..

[31] Wu AH (2006). Serial testing of B-type natriuretic peptide and NTpro-BNP for monitoring therapy of heart failure: the role of biologic variation in the interpretation of results. Am Heart J..

[32] Mueller C (2005). B-type natriuretic peptide for acute dyspnea in patients with kidney disease: insights from a randomized comparison. Kidney Int..

[33] Mehra MR (2004). Obesity and suppressed B-type natriuretic peptide levels in heart failure. J Am Coll Cardiol..

[34] Buffaloe GW, Heineken FG (1983). Plasma volume nomograms for use in therapeutic plasma exchange. Transfusion.

[35] Marenzi G (2001). Circulatory response to fluid overload removal by extracorporeal ultrafiltration in refractory congestive heart failure. J Am Coll Cardiol.

[36] Grodin JL (2019). Prognostic implications of plasma volume status estimates in heart failure with preserved ejection fraction: insights from TOPCAT. Eur J Heart Fail.

