# Supplementary material for: Long-term Prognostic Value of Estimated Plasma Volume in Heart Failure with Preserved Ejection Fraction
Source: Sci Rep. 2019 Oct 7;9:14369. doi: 10.1038/s41598-019-50427-2 (PMC6779908; doi:10.1038/s41598-019-50427-2)
Supplement: Supplementary file 1 — Supplemental Tables [file 41598_2019_50427_MOESM1_ESM.docx]

Supporting Online Materials for

Long-term Prognostic Value of Estimated Plasma Volume in Heart Failure with Preserved Ejection Fraction

Chen-Yu Huang^1,2^; Ting-Tse Lin^3,4*^; Yi-Fan Wu^5^; Fu-Tien Chiang^1^; Cho-Kai Wu^1*^

**Supplemental Table 1. Baseline characteristics of validation cohort**

|  | HFpEF with ADHF |
| --- | --- |
| N | 40 |
| **Baseline** | |
| Age, year | 58.9±13.6 |
| Male, n (%) | 25 (62.5) |
| BMI, kg/m^2^ | 24.8±3.8 |
| BSA (m^2^) | 1.86±0.31 |
| Smoking, n (%) | 13 (32.5) |
| Alcohol, n (%) | 6 (15) |
| **Risk factors** | |
| DM, n (%) | 25 (62.5) |
| HTN, n (%) | 34 (85.0) |
| Dyslipidemia (%) | 23 (57.5) |
| **Comorbidities** | |
| CKD (%) | 16 (40.0) |
| CAD (%) | 28 (70.0) |
| MI (%) | 14 (30.0) |
| CVA (%) | 3 (7.5) |
| PAD (%) | 2 (5.0) |
| NT-proBNP, pg/mL | 453.39±421.56 |
| Diuretics use during hospitalization (mg) | 286.2±97.1 |

BMI: body mass index; BSA: body surface area; DM: diabetes mellitus; HTN: hypertension; CKD: chronic kidney disease; CVA: cerebrovascular accident; CAD: coronary artery disease; MI: myocardial infarction; PAD: peripheral artery disease; NT-proBNP: N-terminal pro B-type natriuretic peptide

**Supplemental Table 2. Correlation between ePVS, ∆ePVS and NT-pro-BNP and diuretics dosage during hospitalization**

|  | NT-Pro-BNP | | Diuretic dosage | |
| --- | --- | --- | --- | --- |
|  | Correlation coefficient | P value | Correlation coefficient | P value |
| ePVS | -0.134 | 0.411 | 0.050 | 0.820 |
| ∆ePVS | -0.468 | 0.039 | 0.526 | 0.019 |

ePVS: estimated plasma volume (Strauss formula); NT-proBNP: N-terminal pro B-type natriuretic peptide
